# Genome-scale CRISPR screening for potential targets of ginsenoside compound K

**DOI:** 10.1038/s41419-020-2234-5

**Published:** 2020-01-20

**Authors:** Yuanyuan Yang, Xiaojian Liu, Shuang Li, Yanhao Chen, Yongxu Zhao, Yuda Wei, Yan Qiu, Yan Liu, Zhihua Zhou, Jun Han, Guohao Wu, Qiurong Ding

**Affiliations:** 1 CAS Key Laboratory of Nutrition, Metabolism and Food Safety, Shanghai Institute of Nutrition and Health, Shanghai Institutes for Biological Sciences, University of Chinese Academy of Sciences, Chinese Academy of Sciences, Shanghai, 200031 P. R. China; 2 CAS-Key Laboratory of Synthetic Biology, Institute of Plant Physiology and Ecology, Shanghai Institutes for Biological Sciences, Chinese Academy of Sciences, Shanghai, 200032 China; 3 Department of General Surgery, Zhongshan Hospital, Fudan University, Shanghai, China; 4 Institute for Stem Cell and Regeneration, Chinese Academy of Sciences, Beijing, 100101 P. R. China

**Keywords:** Macroautophagy, Macroautophagy

## Abstract

Ginsenosides exhibit a large variety of biological activities in maintaining physical health; however, the molecule underpinnings underlining these biological activities remain to be defined. Here, we took a cellular condition that compound K (CK) induces autophagic cell death in HeLa cells, and setup a high-throughput genetic screening using CRISPR technology. We have identified a number of CK-resistant and CK-sensitive genes, and further validated *PMAIP1* as a CK-resistant gene and *WASH1* as a CK-sensitive gene. Compound K treatment reduces the expression of WASH1, which further accelerates the autophagic cell death, highlighting WASH1 as an interesting downstream mediator of CK effects. Overall, our study offers an easy-to-adopt platform to study the functional mediators of ginsenosides, and provides a candidate list of genes that are potential targets of CK.

## Introduction

Ginseng presents a valuable traditional herbal medicine that has been widely used in Asia for millenniums to maintain physical healthy. Its active constituents are ginsenosides, a group of triterpene sapoins, which exhibit a large variety of biological activities, such as anti-tumor^1^, anti-inflammatory^2^, anti-aging^3^, etc. Of more than one-hundred kinds of ginsenosides, compound K (20-O-beta-D-glucopyranosyl-20(S)-protopanaxadiaol, CK) is a major deglycosylated metabolite form, which is absorbed and discovered in circulation^4^. The diverse biological effects of CK is well explored and recognized, and with recent success in constructing “yeast factories” that can produce large amount of CK with high purity^5^, CK presents a promising potential therapeutic agent for many diseases^3,6–8^.

Many signaling pathways and molecular targets have been reported to mediate the diverse biological functions of CK. In tumor cells, CK exhibits a direct cytotoxic and growth-inhibitory effects via pathways including induction of caspase-dependent apoptosis and cell cycle arrest^9^, and increase of mitochondrial disruption. In atherosclerosis and neurodegenerative^10^ conditions, CK treatment can have anti-inflammation and anti-allergic effects, with molecular mechanisms mostly centered around inhibitory functions to NF- κB^7,11,12^ and MAPK pathways^13^, ROS generation^9^, NADPH oxidase activity^5^, and production of inflammatory cytokines^14^. Besides, depending on different cell types studied, many other targets have also been identified, e.g. AMPK^4^, calcium flux^15^, altogether constituting a complicated signaling network that can be influenced by CK treatment.

Despite of all these efforts, there has been no systematic and unbiased analysis of potential downstream targets of CK so far. With recent advances in genome editing technologies, especially the clustered regularly interspaced short palindromic repeats (CRISPR)/CRISPR-associated (Cas) system, genome-wide genetic screening using CRISPR technologies offers a high-throughput, low-cost, and unbiased platform that enables comprehensive study of genetic regulators in a biological condition^16^. We thus wanted to employ this genetic screening approach to identify potential downstream targets of CK. To enable a successful genetic screening, we took advantage of a cellular condition that CK treatment can promote autophagic cell death, which allows a cell-survival-based readout for screening^17^. We then applied genome-wide CRISPR screening technology to this cell model. We have identified a number of “CK-resistant genes”—loss of which can render cells more resistant to CK treatment; and “CK-sensitive genes”—loss of which can make cells more sensitive to CK treatment, offering a useful resource for further exploring the molecular underpinnings to various biological activities of CK. Our study also demonstrates a broadly useful approach for studying the potential molecular targets of traditional herbal medicines.

## Materials and methods

### Cell culture

Human embryonic kidney 293 T cells, human cervix adenocarcinoma HeLa cells, and human hepatocarcinoma HuH-7 cells were purchased from Cell Bank (SIBS, Shanghai), and maintained in DMEM (Dulbecco’s modified Eagle’s medium) supplemented with 10% fetal bovine serum and 1% penicillin/streptomycin. Cells were grown in a humidified CO_2_ incubator at 37 °C.

For chemical treatment, HeLa cells and HuH7 cells were either cultured in a serum-free medium and treated with compound K (5 nM) (A gift form Dr. Zhihua Zhou) for different times as indicated; or cultured in full medium and treated with compound K (5 nM, 5 μM, or 15 μM) for different days as indicated. 3-Methyladenine (3-MA, MedChemExpress, 5142–23–4, 5 mM) was applied 1 day before compound K treatment.

### Lentivirus packaging

To prepare lentiviruses for CRISPR library or a single CRISPR plasmid, HEK293T cells in each 10 cm dish were transfected with 22.5 μg CRISPR plasmids together with 14.7 μg pMDL, 5.7 μg pRev, and 7.9 μg pVSVG packaging plasmids. After transfection, medium with viral particles were collected 48 h and 72 h later and centrifuged at 20,000 r.p.m at 4 °C for 2 h to pellet viral particles. Viral pellets were then resuspended in DMEM at 4 °C overnight and titer was calculated using a PCR-based titration kit (Applied Biological Materials Inc, LV900).

### Genome-wide CRISPR-Cas9 screenings

The genome-wide CRISPR library was purchased from Addgene (#1000000048). Library was amplified following the protocol provided by Addgene, and lentiviruses were prepared and titer was calculated as described above.

For genetic screening, around 3.4 × 10^7^ HeLa cells were infected by the CRISPR lentivirus library to achieve an infection efficiency at around 30%. After virus infection, cells were treated with puromycin (1 μg/ml) for 1 day to eliminate noninfected cells. Cells were then treated with DMSO as control, or compound K (5 nM) for 2 days, allowed to recover for 1 day without drug treatment, and treated with compound K for one more day before harvesting.

Genomic DNA of cells from different groups (DMSO control group and compound K treatment group) was extracted and the sgRNAs were amplified by two-rounds PCR method using KOD DNA polymerase (TOYOBO, KOD-401). In the first round, a total of 2.2 μg genomic DNA (200 ng per PCR reaction; 11 separate reactions for each sample) from each group was used as DNA template; the PCR program used was 94 °C 5 min, 98 °C 30 s, 57 °C 30 s, 68 °C 35 s, 18 cycles. The primers were as following: 5′- TGAAAGTATTTCGATTTCTTGGCTT -3′ and 5′-CGGTGCCACTTTTTCAAGTT-3′. The products (288 bp) were applied in the second round of PCR as template, the PCR program used was 94 °C 5 min, 98 °C 30 s, 57 °C 30 s, 68 °C 35 s, 24 cycles. PCR primers used for amplification were as following: 5′-Barcode + TGAAAGTATTTCGATTTCTTGGCTT-3′, and 5′-Barcode + CGGTGCCACTTTTTCAAGTT-3′. An 8 bp barcode for multiplexing of different biological samples were added at 5′ of each primer. Products (142 bp) were gel-purified and quantified. In total 3.6 μg PCR products from each group were pooled together and subjected for deep sequencing (Illumina HiSeq4000 system).

For data analysis, raw reads of sgRNA were demultiplexed using the FASTX-Toolkit (http://hannonlab.cshl.edu/fastx_toolkit/) and processed to contain only the unique sgRNA sequence. The sgRNA sequences from the GeCKO library were then assembled into a Burrows-Wheeler index using the Bowtie2^18^ build-index function. The sgRNA information from deep sequencing were further aligned to the index using the Bowtie2 aligner. After alignment, the read count statistics for each library sequence was preform using MAGeCK^19^. Significant genes in both positive selection analysis and negative selection analysis are simultaneously identified based on the sgRNA statistical significance of each gene using MAGeCK, using FDR < 0.05.

### Construction of the RGLC3 reporter

The mRFP-GFP-LC3 fragment was constructed using a PCR-based ligation method, which was then inserted between the XbaI and BamHI sites of pLentiCRISPR V2 (Addgene, #52961) to replace the original Cas9 cassette, to obtain the lentivirus-mRFP-GFP-LC3 (Lenti-RGLC3) construct.

Lenti-RGLC3 was then packaged into lentivirus, and used to infect HeLa cells to monitor autophagic influx. The fluorescence was imaged using a fluorescence microscope (Olympus BX53).

### Quantitative RT-PCR analysis

Total RNA was extracted from HeLa using TRIzol reagent (ThermoFisher, 15,596,018) and transcribed with the reverse transcription kit (Takara, RR047A). Quantitative real-time PCR was carried out on the 7900 System(ABI) using SYBR Green supermix (ABI, 4472908). The sequences of primers were as following: 5′-CGGCTACCACATCCAAGGAA-3′ and 5′- GCTGGAATTACCGCGGCT-3′ for human 18S RNA; 5′-AACGACCTCATGTACAGTGC-3′ and 5′-GTGTTAGTACCCCATCTTGTAGG-3′ for human *WASH1* gene. 5′-ACCAAGCCGGATTTGCGATT-3′ and 5′- ACTTGCACTTGTTCCTCGTGG -3′ for human *PMAIP1* gene.

### Generation of CRISPR-Cas9 knockout cell lines

The *WASH1-*KO and *PMAIP1-*KO cell lines were generated by the CRISPR/Cas9 technology. The 20 bp sequences of sgRNA targeting individual genes was inserted into plentiCRISPR V2 and used for lentivirus packaging. The target sequences used are 5′-CAGGCACCATGACTCCTGTG-3′ for human *WASH1*, and 5′-TCGAGTGTGCTACTCAACTC-3′ for human *PMAIP1*. Lentiviruses carrying CRISPR-Cas9 targeting individual genes or empty plentiCRISPR V2 as control viruses were packaged. HeLa cells were then infected and selected with puromycin to remove uninfected cells. Cells were next subjected to genomic DNA extraction for T7EI analysis (NEB, E3321) or protein extraction for western blot to determine gene editing efficiency. Primers used in T7EI analysis for each gene were as follows: 5′- AAATCAATGGTTGTGCACGGTT-3′ and 5′-TAGCAAGCACCTTGTAGGGG-3′ for human *WASH1*;5′-GGACAAAAGCGTGGTCTCTGGCG-3′ and 5′- CTCCTGAACACAGGGGCCCTTG-3′ for human *PMAIP1*.

### Generation of WASH1 overexpressing cell line

For exogenous overexpression of *WASH1* in HeLa cells, human *WASH1* cDNA was amplified, and inserted to the pCDH-EF1 vector (System Biosciences, CD520A-1) between the XbaI and NotI sites, to obtain the pCDH-*WASH1* construct. Primers used to amplify *WASH1* cDNA were as following: 5′- GCTCTAGAATGACTCCTGTGAGGATGCA -3′ and 5′-ACGAGGACGACTGGGAATCGGCGGCCGCTAAACTAT-3′. The pCDH-*WASH1* construct was then packaged into lentivirus, and used to infect HeLa cells for exogenous overexpression.

### Transmission electron microscopy imaging

HeLa cells were fixed overnight with 2.5% glutaraldehyde and 2% paraformaldehyde in cacodylate buffer (0.1 M, pH 7.4). The ultrathin sections were obtained on an ultra cryomicrotome (Ultra Microtome Reichert Ultracut E; Leica Microsystems, Wetzlar, Germany) and were visualized with Joel JEM-1230 transmission electron microscope (TEM).

### Hoechst 33258 staining assay

Hoechst 33258 (ThermoFisher, H3569) staining was performed to capture apoptotic induction of CK to HeLa cells. HeLa cells cultured in serum-free medium were treated with CK (5 nM) or DMSO for 1 or 2 days, before fixed with 4% paraformaldehyde for 30 min at 4 °C. Cells were then stained with Hoechst 33258 solution for 10 min at room temperature and subjected to imaging using a fluorescence microscope (Olympus BX53).

### Flow cytometry assay

HeLa cells cultured in serum-free medium were treated with CK (5 nM) or DMSO for 1 day. Cells and supernatant were then collected and centrifuged, with the cell pellet resuspended in 195 µL binding buffer (Beyotime, C1062S). Cells were later stained with the FITC-Annexin V apoptosis detection kit (Beyotime, C1062S) according to manufacturer’s instructions, and analyzed by flow cytometry using the CytoFLEX S (BECKMAN COULTER).

### Western blot analysis

Protein from cells was extracted by RIPA buffer (Millipore, 20,188) and subjected to regular western procedure. The primary antibodies used in the experiments were alpha-tubulin (Sigma, T6557), β-Actin (CST, 8H10D10), LC3B (Sigma, ABC432), WASH C1 (Sigma, HPA002689), PMAIP1(ABclonal, A9801)

### Statistical analysis

The unpaired, two-tailed Student’s *t*-test was used for experiments with two groups and one-way ANOVA with post-hoc Bonferroni multiple-comparison tests were used for experiments containing more than two groups. All data are represented as means with SEM. ImageJ software was used for quantification of image signals.

## Results

### Compound K treatment induces autophagic cell death

**Fig. 1:**
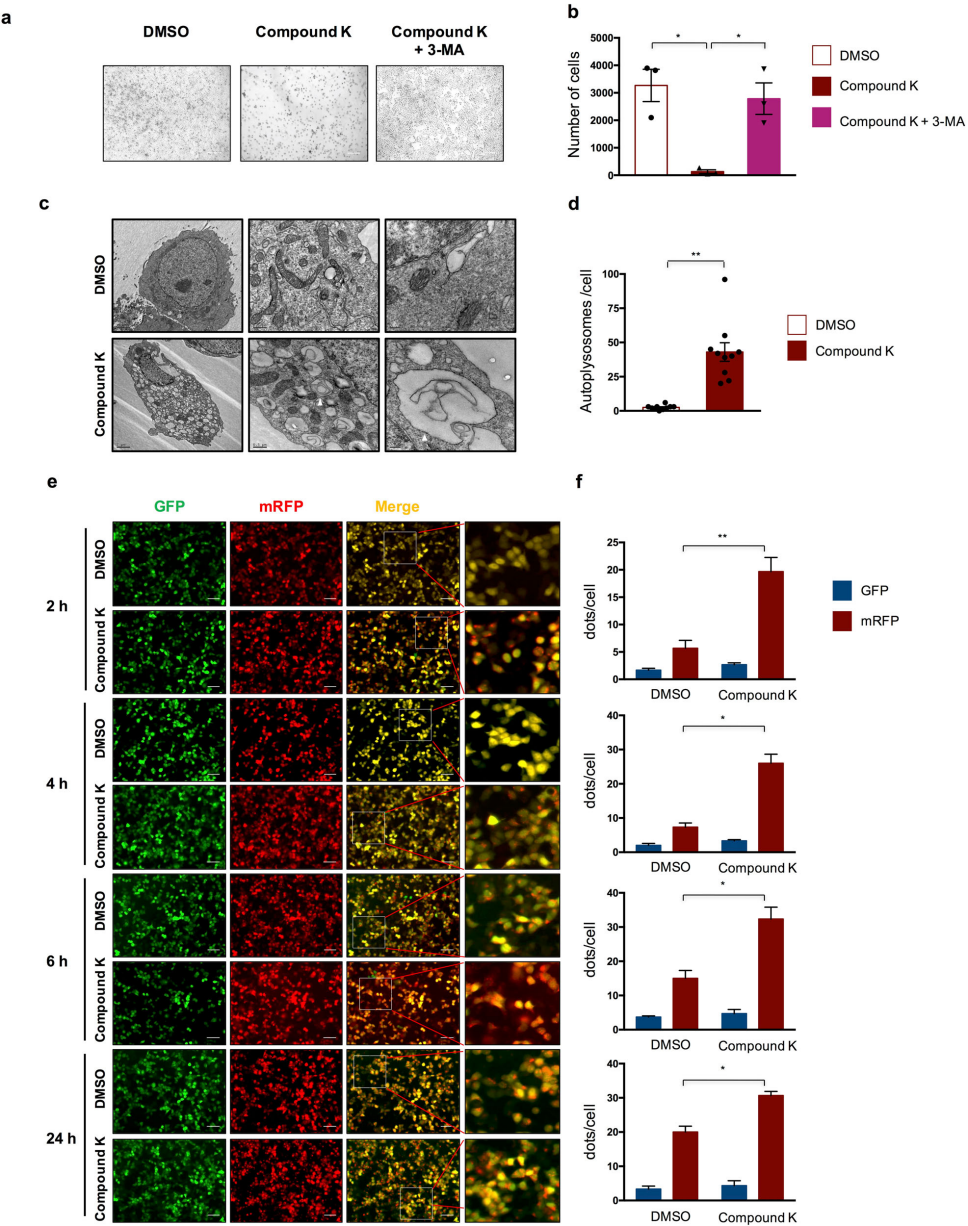
Compound K treatment induces autophagic cell death in HeLa cells. **a** Representative images of cell state after CK (5 nM) or DMSO treatment for 3 days, with or without pretreatment with 3-MA (5 mM) for 1 day. Scale bar = 150 µm. **b** Quantification of cell numbers in each cellular condition as presented in **a**. **c** Representative EM images of cells after CK (5 nM) or DMSO treatment. Scale bar = 2 µm (left), 0.5 µm (middle), 0.2 µm (right). Arrow head, autolysomes. **d** Quantification of autolysosomes in each cellular condition as presented in **c**. **e** Representative images of cellular localization of RGLC3 probe after CK (5 nm) or DMSO treatment for different times as indicated. Scale bar = 150 µm. **f** Quantification of average dots per cell of mRFP and GFP signals in each cellular condition as presented in **e**. Data are represented as means with SEM. *P < 0.05, **P < 0.01.

CK treatment to tumor or nontumor cells can have a direct toxic and growth-inhibitory effect^20–24^, offering an appropriate cellular condition that can be applied in the high-throughput genetic screening via a cell-survival-based readout. We first evaluated the effects of CK treatment to HeLa cell line, which is a cervical tumor cell line, and has been widely used in tumor studies. CK treatment at a low concentration (5 nM) for 3 days in a FBS starvation medium induced a massive cell death (Fig. 1a, b, Fig. S1a, b); whereas the cell death can be reversed by pretreating the cells with 3-methyladenine (3-MA), which is a selective inhibitor to class III phosphatidylinositol 3-kinase (PtdIns3K) and can also block the formation of autophagosome^25^ (Fig. 1a, b). The remaining cells after CK treatment also displayed significantly more autolysosomes, as compared to control cells, under the transmission electron microscope (TEM) (Fig. 1c, d). Altogether, these results suggest autophagic cell death induced by CK treatment.

We further confirmed this phenomenon by using the fluorescent probe, GFP-mRFP-LC3, to evaluate autophagic flux^26^. The membrane-bound microtubule associated protein 1 light chain 3 pro (LC3) is fused with green and red fluorescent proteins. GFP, but not mRFP, is unstable in acidic environment, and undergoes degradation in autolysomes. The GFP/mRFP ratio can therefore be calculated to estimate autophagic flux. HeLa cells were infected by lentivirus-mRFP-GFP-LC3 (Lenti-RGLC3), and then was treated with DMSO or CK for 1–24 h. Consistent with previous findings, the induction of autophagy was more obvious and the autophagic flux was significantly accelerated in CK-treatment groups, as compared to control cells (Fig. 1e, f). Further tests in full medium containing FBS also demonstrated the induction of autophagy and cell death upon CK treatment, albeit at a much higher concentration (Fig. S1c, d). Additionally, similar phenomenon was observed in HuH7 cells after CK treatment (Fig. S2). Taken together, these results indicate that CK treatment induces autophagic cell death in HeLa as well as HuH7 cells.

### High-throughput screenings identify potential CK-sensitive and CK-resistant genes

**Fig. 2:**
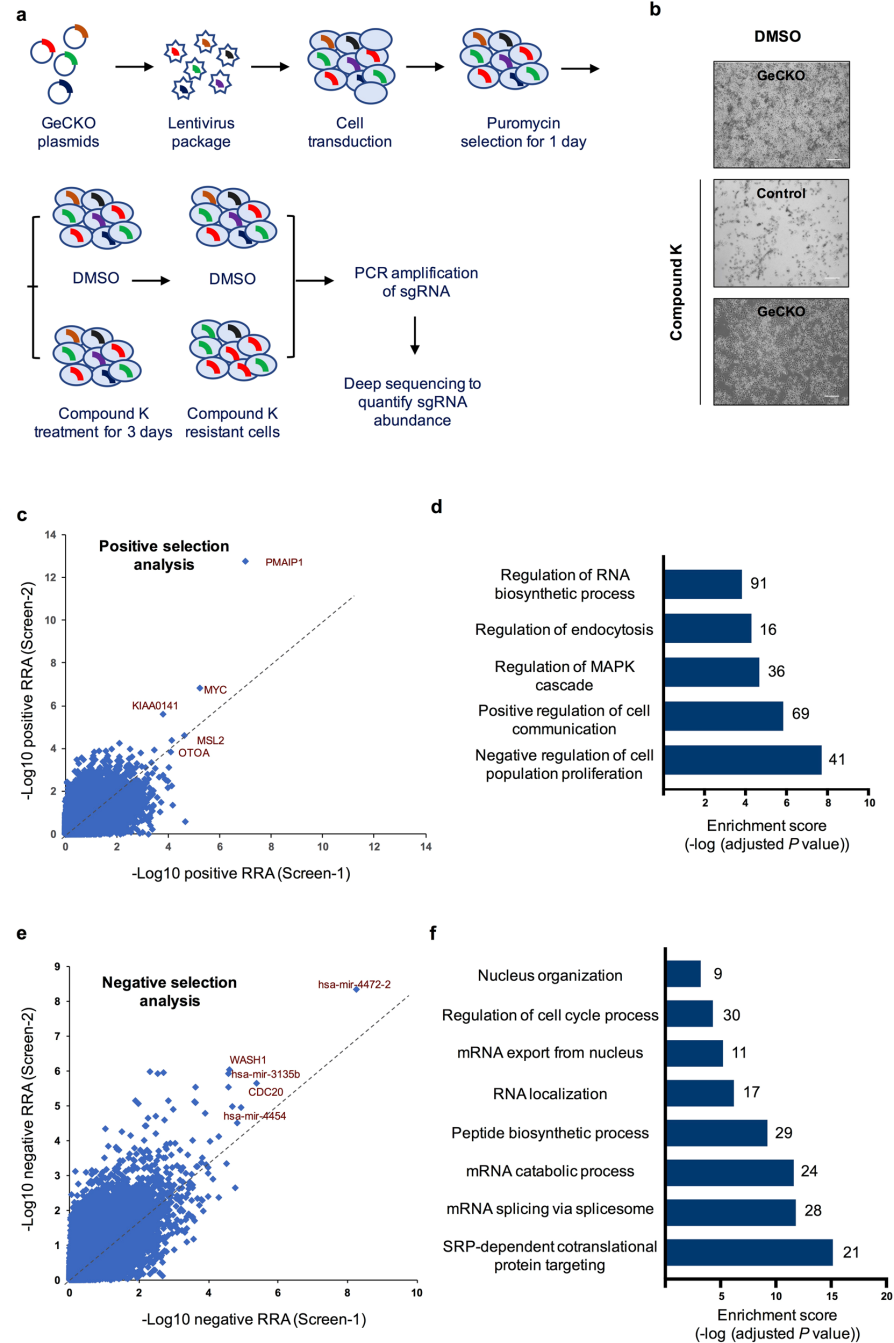
High-throughput screenings identify CK-resistant and CK-sensitive genes. **a** Schematic view of the experimental setup in the high-throughput genetic screenings. **b** Representative images of cell state at each treatment condition as indicated. Scale bar = 150 µm. **c** Scatterplot showing significant genes in positive selection analysis. **d** Gene Oncology and pathway analysis of positive selection genes. **e** Scatterplot showing significant genes in negative selection analysis. **f** Gene Oncology and pathway analysis of negative selection genes. RRA, Robust Ranking Aggregation score generated in the MAGeCK algorithm.

To further explore the potential targets of CK, we setup a genome-wide genetic screening by using the GeCKO library^16^. GeCKO library lentiviruses were applied to infect HeLa cells at an efficiency of around 30%, to ensure that most of the cells were infected by one virus. Cells were then selected with puromycin to remove uninfected cells, before further treatment with CK or DMSO control. Cells infected with control virus were also treated with CK and used as a negative control (Fig. 2a). Consistent with previous results, CK treatment induced a massive cell death. However, there are significantly more cells left in GeCKO virus-infected cells, as compared to cells infected with control viruses (Fig. 2b). The survival cells in GeCKO group were then grown up, and genomic DNA was further isolated for deep sequencing to retrieve the gRNA information. Two independent screenings were performed, and cells infected with GeCKO library and treated with DMSO control were included for gRNA diversity comparison.

We did both positive selection analysis and negative selection analysis. Positive selection analysis aims to identify gRNAs that are more enriched in survival cells after CK treatment, as compared to the DMSO control group, which enables the discovery of CK-resistant genes—loss of which can render cells more resistant to CK-induced cell death. Whereas negative selection analysis is intended to identify gRNAs that are more depleted in survival cells after CK treatment, emphasizing the discovery of CK-sensitive genes—loss of which can make cells more sensitive to CK-induced cell death. In both analyses, using FDR < 0.05 as a cutoff, we have identified 584 CK-resistant genes in positive selection analysis (Table. S1), and 368 CK-sensitive genes in negative selection analysis (Table. S2).

In CK-resistant genes, we noticed that several of the top genes are functionally related to cell growth and cell survival (Fig. 2c). For example, *PMAIP1*, encoding phorbal-12-myristate-13-acetate-induced protein 1, promotes activation of caspases and apoptosis;^27^ *KIAA0141*, also called *DELE1*, encoding DAP3-binding cell death enhancer 1, is essential for the induction of death receptor-mediated apoptosis through regulation of caspase activation^28^. Analysis of the 584 CK-resistant genes revealed significant enrichment in pathways in negative regulation of cell population proliferation (41 genes), positive regulation of cell communication (69 genes), regulation of MAPK cascade (36 genes), etc (Fig. 2d).

In CK-sensitive genes, several miRNAs stood out in the top genes (Fig. 2e). More interestingly, analysis of the 368 CK-sensitive genes revealed enriched pathways related to RNA process, including mRNA splicing, mRNA catabolic, RNA localization, mRNA export from nucleus (Fig. 2f). The molecular underpinnings behind this phenomenon are worth further investigation.

### *PMAIP1* knockout cells are resistant to autophagic cell death induced by compound K treatment

**Fig. 3:**
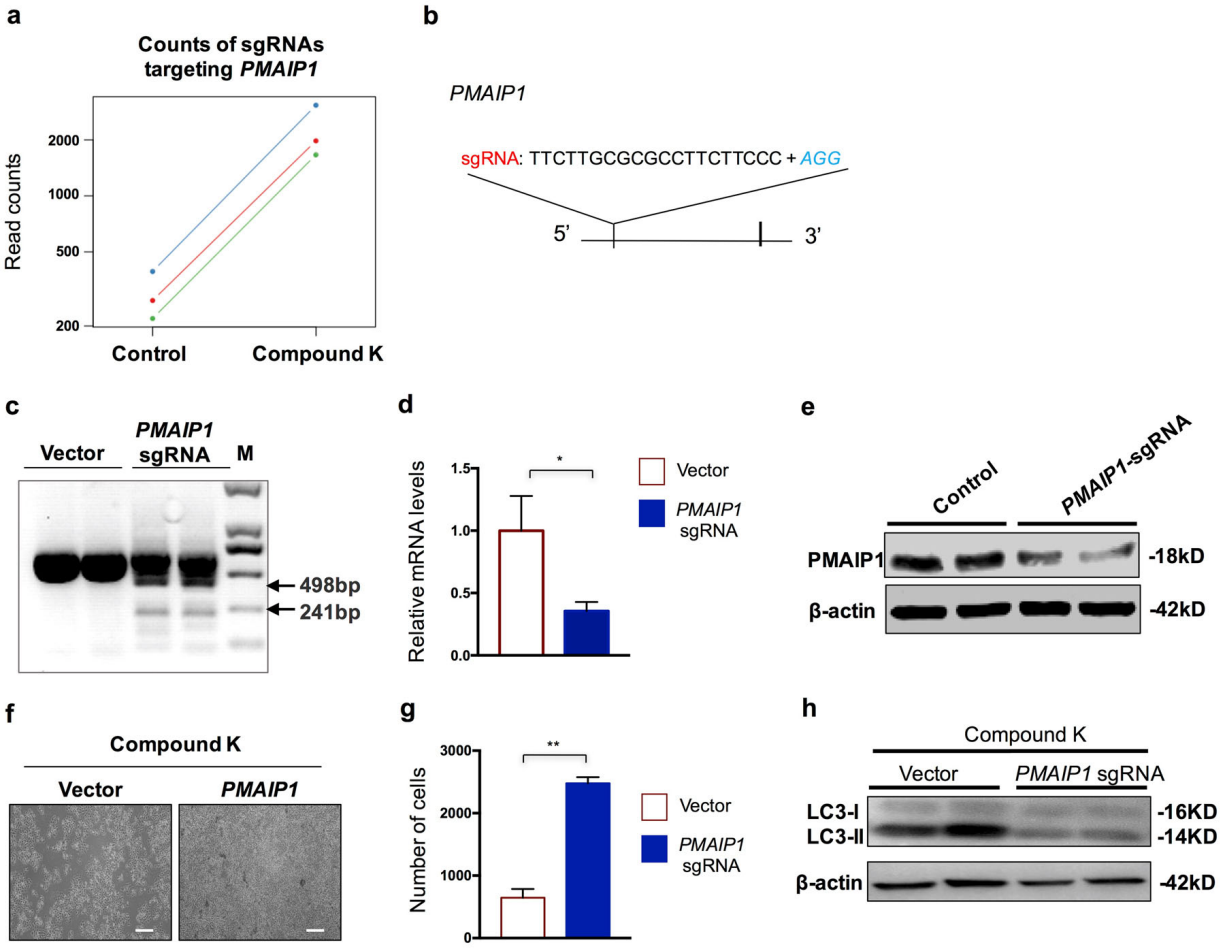
PMAIP1 is a CK-resistant gene. **a** Read counts of sgRNAs targeting *PMAIP1* in control and CK-treated groups. **b** Illustration of the sgRNA applied to deplete *PMAIP1* in validation experiments. **c** Genome editing activity as assessed by T7E1 assay of sgRNA targeting *PMAIP1*. **d** Relative mRNA expression level of *PMAIP1* in control and *PMAIP1* sgRNA-treated cells. **e** Analysis of the protein level of PMAIP1 in control and *PMAIP1* sgRNA-treated cells. **f** Representative images of cell state after CK (5 nM) treatment for 3 days. Scale bar = 150 µm. **g** Quantification of cell numbers in each cellular condition as presented in **f**. **h** Analysis of the LC3 protein level in control and *PMAIP1* sgRNA-treated cells after CK treatment for 1 h. Data are represented as means with SEM. *P < 0.05, **P < 0.01.

We further did validation of these top hits in both analyses. *PMAIP1*, also named *NOXA*, is a top hit in positive selection analysis. All three gRNAs targeting *PMAIP1* displayed a significant enrichment in survival cells after CK treatment (Fig. 3a). *PMAIP1* encodes a BH3-containing mitochondrial protein, which can disrupt mitochondrial outer membrane integrity and cause the apoptosis^29^. To further validate the functional involvement of PMAIP1 in cell death caused by CK treatment, we simply targeted *PMAIP1* via CRISPR-Cas9 technology in HeLa cells (Fig. 3b). CRISPR targeting resulted in a clear cutting at the *PMAIP1* genomic locus as revealed by the T7 endonuclease 1 (T7E1) assay (Fig. 3c), and subsequently significant reduction in mRNA expression due to nonsense mediated decay (Fig. 3d), and protein expression (Fig. 3e). Consistent with the screening result, *PMAIP1*-targeted cells showed significant resistance in cell death in response to CK treatment (Fig. 3f, g). Additionally, further analysis demonstrated a reduced autophagy induction process in *PMAIP1*-targeted cells, as revealed by less lipidated LC3 (Fig. 3h). However, CK treatment did not alter the expression of PMAIP1 at mRNA or protein level (Fig. S3).

### *WASH1* knockout cells are more sensitive to autophagic cell death induced by compound K treatment

**Fig. 4:**
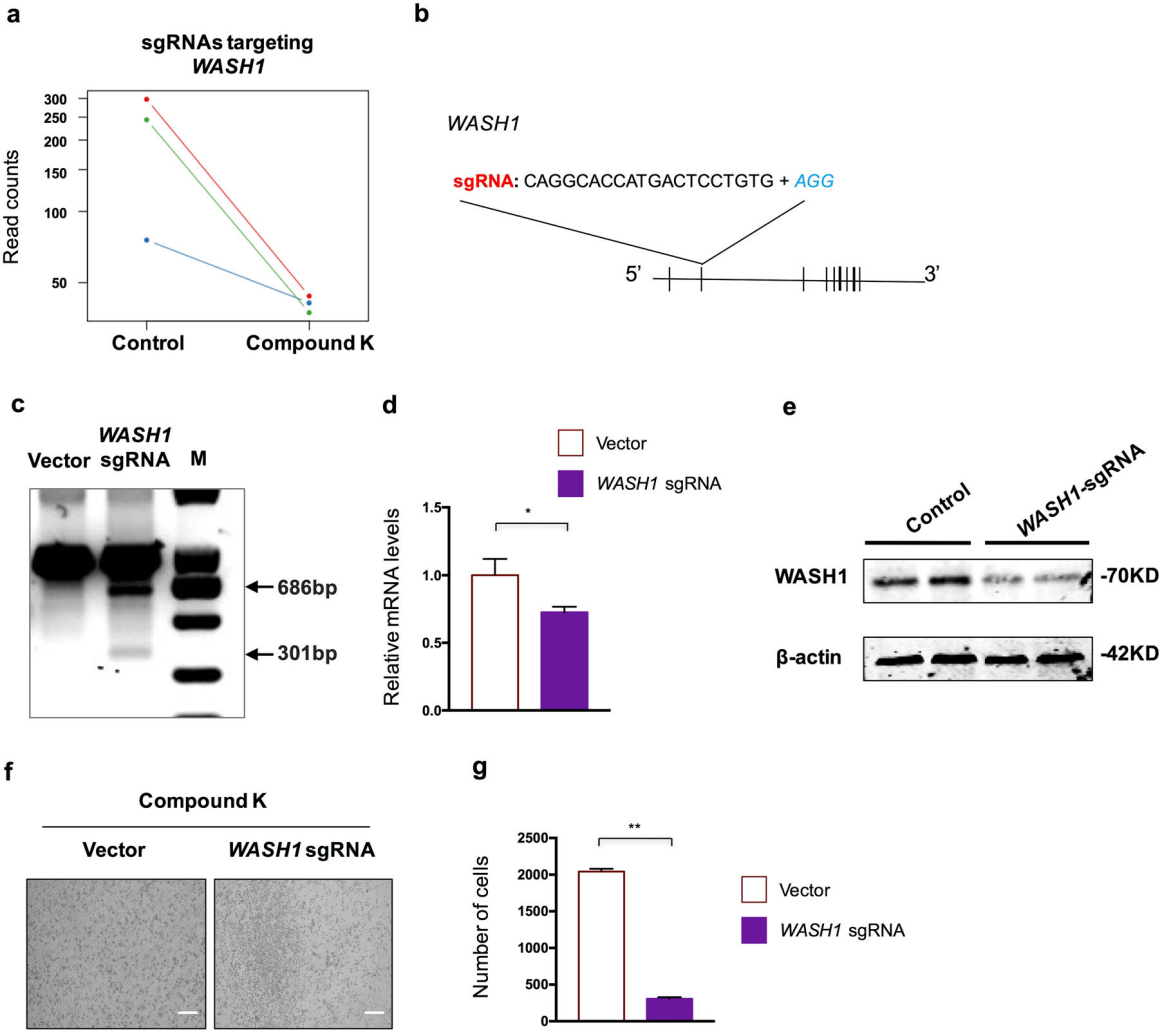
WASH1 is a CK-sensitive gene. **a** Read counts of sgRNAs targeting *WASH1* in control and CK-treated groups. **b** Illustration of the sgRNA applied to deplete *WASH1* in validation experiments. **c** Genome editing activity as assessed by T7E1 assay of sgRNA targeting *WASH1*. **d** Relative mRNA expression level of *WASH1* in control and *WASH1* sgRNA-treated cells. **e** Analysis of the protein level of WASH1 in control and *WASH1* sgRNA-treated cells. **f** Representative images of cell state after CK (5 nM) treatment for 1 day. Scale bar = 150 µm. **g** Quantification of cell numbers in each cellular condition as presented in **f**. Data are represented as means with SEM. *P < 0.05, **P < 0.01.

We next focused on one of top hits in negative selection analysis. *WASH1*, encoding WASH complex subunit 1, which is previously known to function during endosome sorting and endocytic trafficking^30–32^, and also in regulation of autophagy independently from its role in endosomal sorting^33^. All three gRNAs targeting *WASH1* displayed a consistent depletion in survival cells after CK treatment, ranking *WASH1* as a significant negative selection gene (Fig. 4a). To further validate the role of *WASH1* in CK-induced cell death, CRISPR technology was adopted to target *WASH1* in HeLa cells (Fig. 4b). CRISPR targeting led to an obvious cutting at the *WASH1* genomic locus as revealed by the T7 endonuclease 1 (T7E1) assay (Fig. 4c), resulting in significant decrease in mRNA level of *WASH1* (Fig. 4d), and elimination of major WASH1 proteins (Fig. 4e). Importantly, when *WASH1*-targeted cells were subjected to CK treatment, these cells displayed a hypersensitive reaction to CK, with dying of the majority of cells after only 1-day treatment (Fig. 4f, g).

**Fig. 5:**
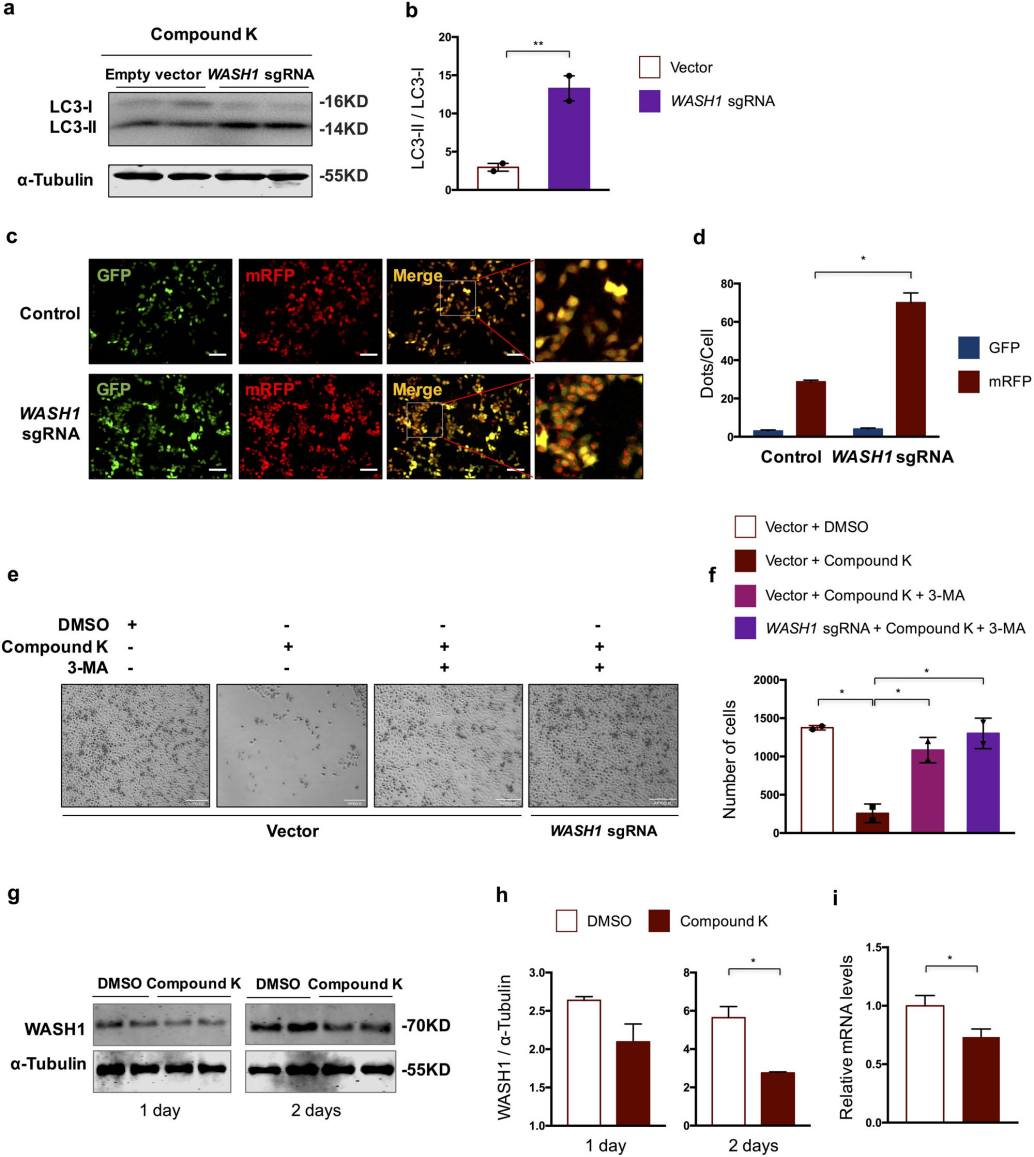
WASH1 is a mediator of CK-induced autophagic cell death. **a** Analysis of the LC3 protein level in control and *WASH1* sgRNA-treated cells after CK treatment for 1 h. **b** Quantification of LC3-II/LC3-I in **a**. **c** Representative images of cellular localization of RGLC3 probe in control and *WASH1* sgRNA-treated cells after CK treatment for 1 h. Scale bar = 150 µm. **d** Quantification of average dots per cell of RFP and GFP signals in each cellular condition as presented in **c**. **e** Representative images of cell state at each condition as indicated. Scale bar = 8 µm. **f** Quantification of cell numbers in each cellular condition as presented in **e**. **g** Analysis of the protein level of WASH1 after DMSO or CK treatment for 1 or 2 days. **h** Quantification analysis of WASH1 protein level in **g**). **i** Relative mRNA expression level of *WASH1* after DMSO or CK treatment for 2 days. Data are represented as means with SEM. *P < 0.05, **P < 0.01.

Further analysis demonstrated a stronger autophagy induction process in *WASH1*-targeted cells, as revealed by more lipidated LC3 (Fig. 5a, b), accelerated autophagic influx (Fig. 5c, d), in comparison to control cells, and the reverse of cell death by pretreatment with 3-MA (Fig. 5e, f). These results altogether demonstrate that WASH1 is a negative regulator in CK-induced autophagic cell death.

We next examined whether CK treatment affects WASH1 expression in HeLa cells. Indeed, we found decreased WASH1 expression in HeLa cells upon CK treatment, both at the protein level (Fig. 5g, h) and at the mRNA level (Fig. 5i). These results indicated that CK treatment led to decreased cellular WASH1 expression, which releases the negative control of autophagy and leads to autophagic cell death.

To examine whether recovery WASH1 expression is enough to rescue the CK-induced autophagic cell death, we increased WASH1 expression in cells via exogenous lentiviral expression (Fig. S4a, b). However, we did not observe significant protective effect after WASH1 overexpression (Fig. S4c, d), suggesting that other than the WASH1 pathway, there are also other pathways involved in CK-induced cell death.

## Discussion

Given the promising therapeutic potentiality of compound K as well as other ginsenosides^1–3^, it remains an intriguing question regarding the specific molecules and pathways that mediate the variety effects of a certain ginsenoside. Here, we addressed this question by performing a CRISPR-based high-throughput genetic screening in CK-treated HeLa cells, and identified a number of CK-resistant and CK-sensitive genes. We further validated *PMAIP1* as a CK-resistant gene and *WASH1* as a CK-sensitive gene, pointing out *WASH1* as an interesting downstream mediator of CK, which is involved in CK-induced autophagic cell death.

In this study, we took advantage of a cellular condition that CK treatment induces autophagic cell death, offering a cell-survival-based readout for screening. While cell survival as a readout is often adopted and favorable in setting up high-throughput genetic screenings, this presents an extreme cellular condition, thus cannot represent many other functional effects of CK treatment. In the positive selection analysis, a number of candidate genes are involved in regulation of cell growth or apoptosis^28,34–36^, which is in fact common in a cell survival based screening, and cannot being simply considered to be specific to CK treatment. However, there are also many other genes that are not functionally connected to cell survival that have being identified in the screening. It is interesting to see that genes enriched in the regulation of endocytosis and RNA biosynthetic process have been identified in positive selections, and genes enriched in many aspects of RNA processing and even nucleus organization have been identified in the negative selection. In fact, in the further validation experiments, we have confirmed *WASH1* as a CK-sensitive gene. CK treatment reduces the expression of WASH1 at both the transcriptional and translational levels, which further accelerates the autophagic cell death, with *WASH-1* depleted cells becoming hypersensitive to CK treatment. These results are consistent with the previous discovery that WASH1 is a negative regulator of autophagy. However, WASH1 is also an important regulator in endocytosis^30–32^, whether CK treatment can also affect cell endocytic trafficking and cargo sorting via WASH1 warrants further investigation. The presentation of RNA processing related genes in both positive and negative selection analyses, suggest that CK treatment may also affect RNA biology directly or indirectly, raising an interesting direction for further functional pursuing of ginsenosides.

Taken together, our study, for the first time, has established a high-throughput genetic screening, by which a number of CK-resistant and CK-sensitive genes have been identified. We have also confirmed the involvement of two genes, *PMAIP1* and *WASH1*, in CK treatment induced autophagic cell death in HeLa cells. Many other targets will also need to be further validated before conclusions can be made. Nonetheless, our study reveals interesting targets of compound K, and offers one easy-to-adopt platform to study the potential functional mediators of ginsenosides.

## Supplementary information

Supplemental Figure Legends

Supplemental Figure 1

Supplemental Figure 2

Supplemental Figure 3

Supplemental Figure 4

Supplemental Table 1

Supplemental Table 2
