# Supplementary material for: Genome-scale CRISPR screening for potential targets of ginsenoside compound K
Source: Cell Death Dis. 2020 Jan 20;11(1):39. doi: 10.1038/s41419-020-2234-5 (PMC6971025; doi:10.1038/s41419-020-2234-5)
Supplement: Supplementary file 1 — Supplemental Figure Legends [file 41419_2020_2234_MOESM1_ESM.docx]

**Supplemental Figure legends**

**Figure S1. Compound K treatment induces autophagic cell death in HeLa cells.** (a) HeLa cells cultured in serum-free medium were treated with CK (5 nM) or DMSO for 1 or 2 days before staining with Hoechst 33258. Cells with nuclear condensation and/or fragmentation (indicating of cell apoptosis) were marked with arrows. One of three independent experiments. Scale bar = 150 µm. (b) Flow cytometry apoptosis analysis of HeLa cells after treatment with CK (5 nM) or DMSO for 1 day in serum-free medium. One of three independent experiments. (c) HeLa cells cultured in full medium were treated with CK (5 nM, 5 µM, 15 µM) or DMSO for different days as indicated before imaging. One of three independent experiments. Scale bar = 150 µm. (d) Representative images of cellular localization of RGLC3 probe after treatment with CK (5 nM, 5 µM, 15 µM) or DMSO for 1 day in full medium. One of three independent experiments. Scale bar = 150 µm.

**Figure S2. Compound K treatment accelerates autophagic flux in HuH7 cells.** HuH7 cells cultured in serum-free medium were treated with CK (5 nM) or DMSO for different times as indicated before imaging. (a) Representative images of HuH7 cells after treatment with CK or DMSO for 3 days. One of three independent experiments. Scale bar = 150 µm. (b) Representative images of cellular localization of RGLC3 probe. Scale bar = 150 µm. One of three independent experiments. (c) Quantification of average dots per cell of mRFP and GFP signals in each cellular condition as presented in (a). n = 10, one of three independent experiments. Data are represented as means with SEM. ** P < 0.01, *** P < 0.001.

**Figure S3. CK treatment causes no effect in PMAIP1 expression in HeLa cells.** (a) Relative mRNA expression level of *PMAIP1* after DMSO or CK treatment for 2 days. n = 3, one of three independent experiments. (b) Analysis of the protein level of PMAIP1 after DMSO or CK treatment for 1 or 2 days. Data are represented as means with SEM.

**Figure S4.** **Exogenous *WASH1* expression causes no significant effect to CK-induced cell death.** (a) Analysis of the protein level of WASH1 in control and *WASH1* overexpressing cells. (b) Quantification of WASH1 level in (a). (c) Representative images of cell state of control and *WASH1* overexpressing cells after CK treatment for 3 days. Scale bar = 150 μm. (d) Quantification of cell numbers in each cellular condition as presented in (c). n = 3, one of three independent experiments. Data are represented as means with SEM. * P < 0.05.

**Table S1. Genes identified in positive selection analysis.**

**Table S2. Genes identified in negative selection analysis.**
