# Supplementary figures and images for: Genome-scale CRISPR screening for potential targets of ginsenoside compound K
Source: Cell Death Dis. 2020 Jan 20;11(1):39. doi: 10.1038/s41419-020-2234-5 (PMC6971025; doi:10.1038/s41419-020-2234-5)

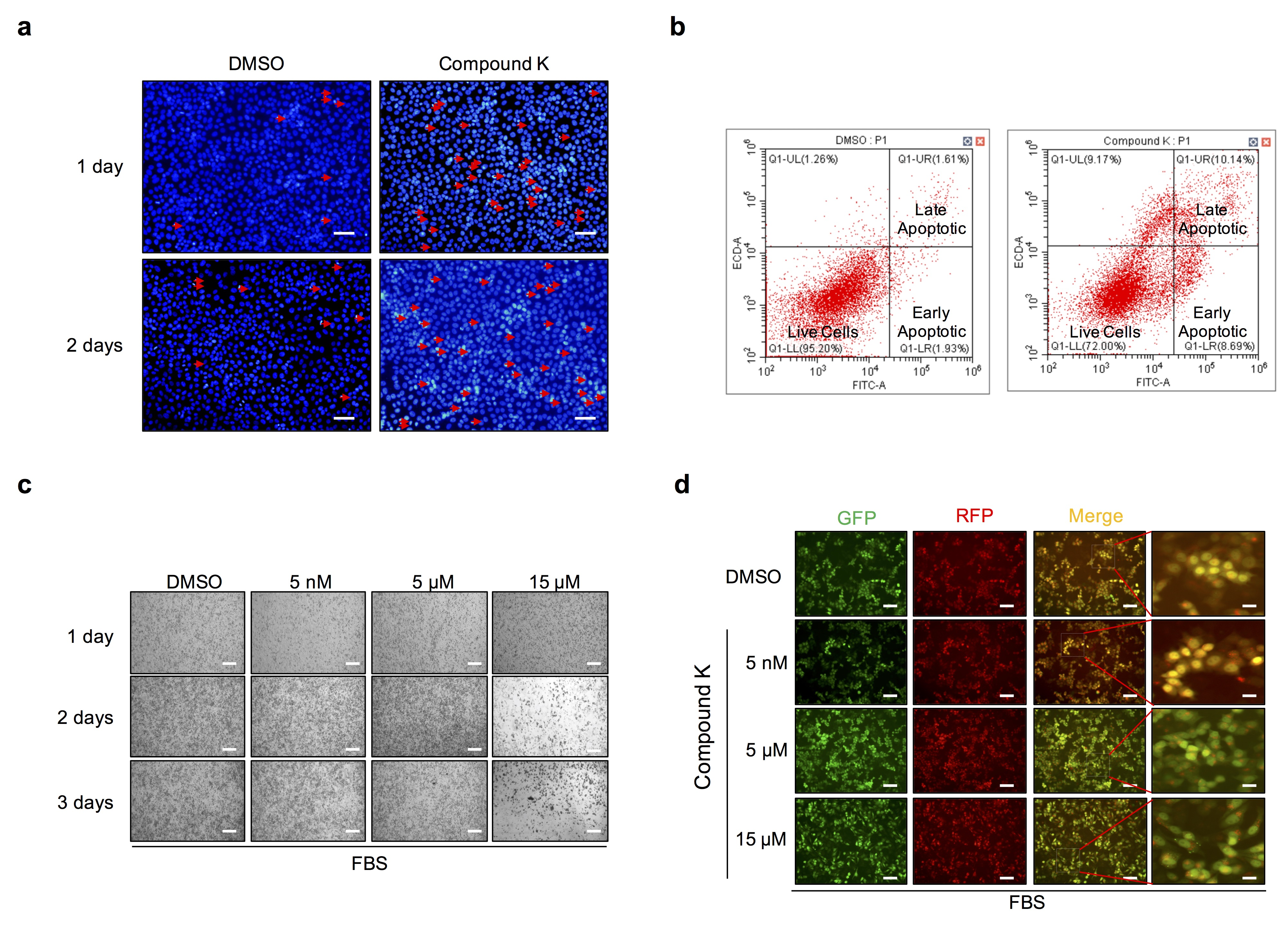

Supplement: Supplementary file 2 — Supplemental Figure 1 [file 41419_2020_2234_MOESM2_ESM.png]

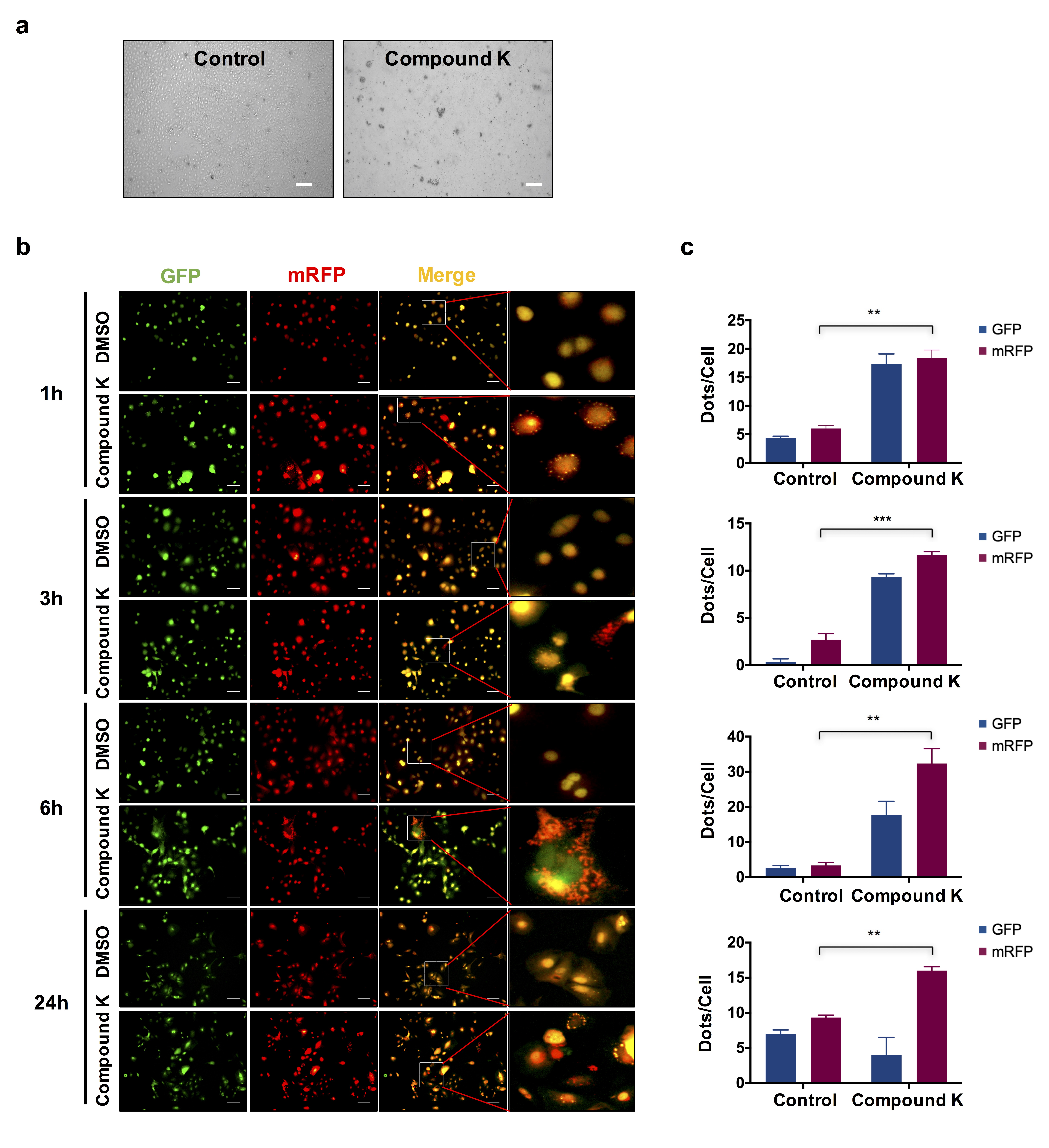

Supplement: Supplementary file 3 — Supplemental Figure 2 [file 41419_2020_2234_MOESM3_ESM.png]

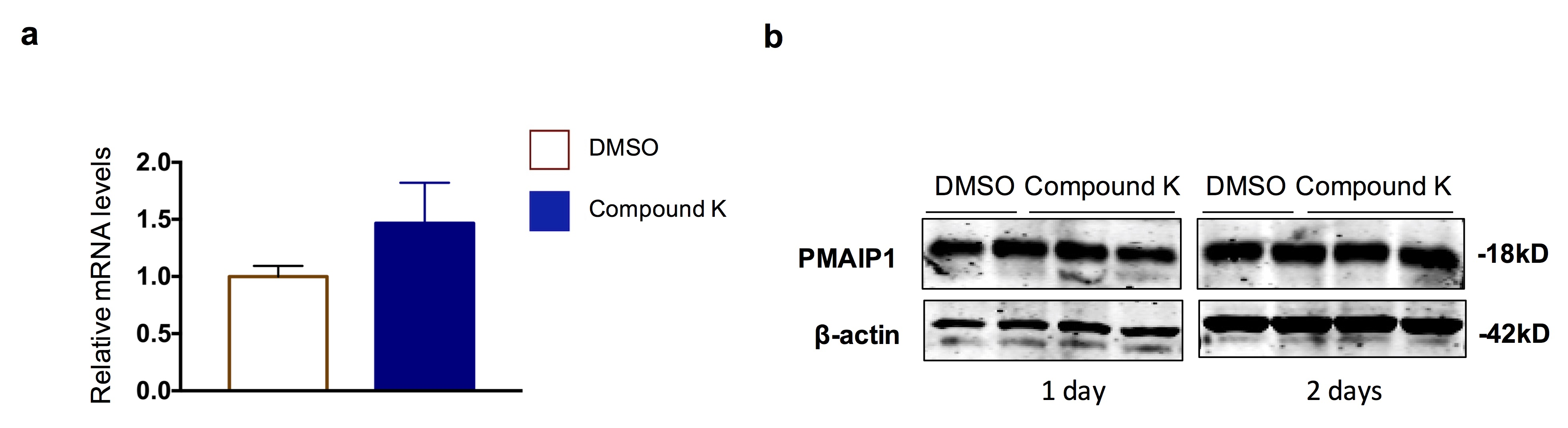

Supplement: Supplementary file 4 — Supplemental Figure 3 [file 41419_2020_2234_MOESM4_ESM.png]

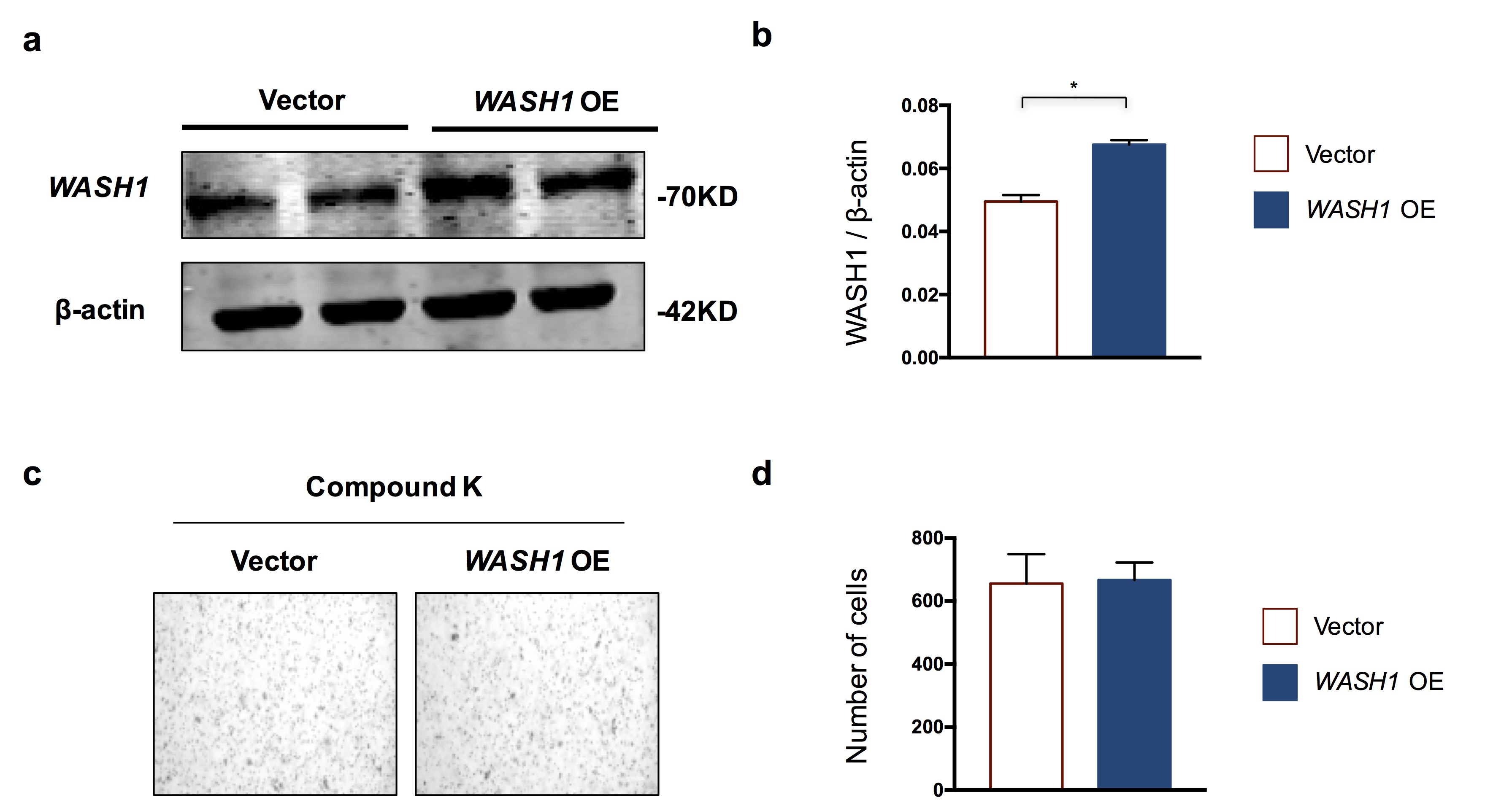

Supplement: Supplementary file 5 — Supplemental Figure 4 [file 41419_2020_2234_MOESM5_ESM.png]
